# Association between psychological resilience and body mass index in a community‐based population: A cross‐sectional study

**DOI:** 10.1002/osp4.761

**Published:** 2024-05-11

**Authors:** Nan Zheng, Mansi Zhuang, Yanan Zhu, Yu Wang, Meijie Ye, Yasi Zhang, Yiqiang Zhan

**Affiliations:** ^1^ Futian Institute of Health Education Shenzhen China; ^2^ Department of Epidemiology School of Public Health (Shenzhen) Sun Yat‐Sen University Shenzhen China; ^3^ Institute of Environmental Medicine Karolinska Institutet Stockholm Sweden

**Keywords:** body mass index, obesity, psychological resilience, stress

## Abstract

**Background and Objective:**

While earlier studies have focused on the relationship between stress and obesity, there was a gap in understanding the potential impact of positive psychological factors, such as resilience, on obesity. By investigating the role of psychological resilience with obesity, this study aimed to address this gap and tackle obesity through a positive psychological framework.

**Methods:**

Participants consisted of 2445 community residents from Shenzhen, China, with a mean age of 41.09 ± 13.72 years, comprising 846 males and 1599 females. Psychological resilience was measured using the Brief Resilience Scale; gender, age, marital status, education level, smoking status, alcohol consumption, frequency of physical exercise, and perceived stress were considered potential confounding factors. The relationship between psychological resilience and body mass index (BMI) was examined through multiple linear regression and logistic regression analyses.

**Results:**

The participants had an average psychological resilience score of 3.46 (standard deviation [SD] = 0.62) and an average BMI of 22.59 (SD = 3.35), with 104 individuals (4.3%) identified with obesity. In the fully adjusted multiple linear regression model, a higher psychological resilience score was associated with a higher BMI (*β* = 0.507, 95% CI:0.283, 0.731). In the logistic regression model, higher psychological resilience scores were linked to increased obesity risk, with a more significant association observed among males (odds ratio [OR] = 2.169, 95% CI:1.155, 4.073), while psychological resilience acted as a protective factor against underweight among females (OR = 0.528, 95% CI:0.376, 0.816).

**Conclusion:**

The study demonstrated a significant link between higher psychological resilience and elevated BMI, emphasizing the complex relationship between psychological fortitude and weight management. Interventions targeting socioeconomic status, education, lifestyle habits, and physiological well‐being might offer a promising strategy for enhancing psychological resilience and promoting healthier weight. Emphasizing self‐efficacy and coping skills at the individual level could contribute to balanced weight and comprehensive health outcomes, addressing the global challenge of obesity.

## Graphical abstract

Higher levels of psychological resilience are associated with elevated BMI, whereas lower resilience scores are linked to underweight, especially among females. This highlights the complex interplay between psychological resilience and weight management, emphasizing the necessity for targeted interventions to address the diverse impact of psychological factors on weight outcomes.

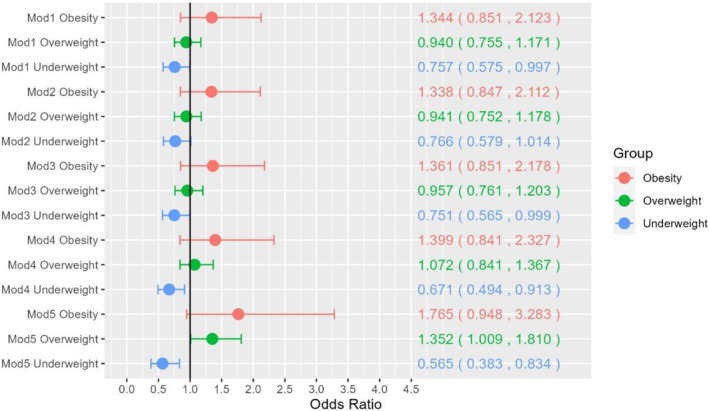

## 1 INTRODUCTION

The rising prevalence of obesity presented a major public health challenge,^1^, ^2^, ^3^ with psychological factors playing a crucial role in its development.^4^, ^5^, ^6^, ^7^ Over the past 3 decades, there has been a steady rise in average body mass index (BMI) and waist circumference among adults,^2^, ^4^ highlighting the intricate relationship between obesity and stress.^5^, ^6^, ^7^ Previous research has shown that obesity itself could induce a state of stress and indicated a positive correlation between stress and weight gain.^8^ Stress impacted cognitive processes, eating behaviors, physical activity, sleep patterns, and hormonal regulation.^9^, ^10^, ^11^ Additionally, exposure to high‐calorie food images could activate specific brain regions, leading to increased food intake in stressed individuals.^8^ In both the Chinese and American contexts, stress might trigger behaviors such as overeating and disrupt activity patterns.^12^, ^13^, ^14^, ^15^, ^16^ The stress response system could release glucose into the bloodstream, potentially leading to the accumulation of body fat.^17^ Obesity and overweight have also been linked to negative psychological effects, impacting emotional and cognitive functions.^18^ This complex relationship highlighted the intricate interplay between psychological and physiological factors in the context of obesity.^18^

The existing body of research has consistently demonstrated a negative association between psychological resilience and stress, underscoring the capacity of individuals to return to a state of normal functioning following significant adversity.^19^, ^20^, ^21^, ^22^ Resilience to stressors explained why individuals could maintain a positive outlook in the aftermath of challenging experiences.^23^ Interventions designed to bolster resilience in individuals who have encountered stress have the potential to enhance their ability for positive adaptation.^24^ Furthermore, evidence suggests that resilience may serve to mitigate the detrimental health impacts of stress.^25^ Despite these findings, the influence of psychological resilience on BMI has received limited attention in the literature.^26^

In light of this, this study aimed to examine the relationship between psychological resilience and BMI among community residents in Shenzhen, China.

## 2 MATERIALS AND METHODS

### 2.1 Study participants

The study employed a random sampling methodology to recruit community residents from the Futian District in Shenzhen, China. This district encompassed 104 administrative communities, which were utilized as natural clusters for the sampling process. Ten percent of these clusters (10 communities) were randomly selected, and administrative officials from eight communities provided consent for participation. The study included individuals who were citizens or permanent residents of the local area, aged 18 years or older, and living in the selected communities. Specifically, the inclusion criteria encompassed individuals registered as Shenzhen citizens, excluding those who had resided outside Shenzhen for more than 6 months, as well as non‐registered Shenzhen citizens with temporary residence permits and a minimum residency duration of 6 months in Shenzhen. One adult member for each of the 3014 households in the selected communities was invited to participate. Ultimately, 3014 individuals were invited, and 2445 participants aged 18 years and older completed the questionnaires.

This study was approved by the Ethical Review Board of the School of Public Health (Shenzhen), Sun Yat‐Sen University.

### 2.2 Resilience score

The research applied the Brief Resilience Scale (BRS) developed by Smith et al., consisting of six items. The scale consisted of three positive vocabulary items (1, 3, 5) and three negative vocabulary items (2, 4, 6), rated on a five‐point Likert scale. Various versions of BRS have been well‐validated in the Chinese population^27^, ^28^ and the present study (Table S1 and Figure S1). The score was calculated as the average of the six items with higher scores indicating a higher level of resilience.^29^, ^30^, ^31^

### 2.3 Stress score

The Perceived Stress Scale (PSS) is a widely used stress assessment scale in the world. The scale used in the present study is the Chinese version of the PSS (CPSS) translated and revised in 2003.^32^, ^33^ All of the 14 items were weighted by the two factors of feeling out of control and feeling of tension. The dimension of tension included items 1, 2, 3, 8, 11, 12, and 14 for positive scores, while the dimension of sense of loss included items 4, 5, 6, 7, 9, 10, and 13 for negative scores. The total score was the sum of 14 items, ranging from 14 to 70 points. The higher score indicated a higher level of perceived stress.

### 2.4 BMI and obesity

BMI was calculated by dividing weight in kilograms by the square of height in meters. According to the Chinese classification standard for obesity,^1^ BMI was classified into four categories: underweight, normal weight, overweight, and obese. This study used criteria for Asian populations to define overweight (BMI ≥24.0 kg/m^2^) and obesity (BMI ≥28.0 kg/m^2^).

### 2.5 Statistical analysis

Data analysis was performed using R version 4.2.2 using packages *ltm* and *nnet*. Frequency was used to describe count data, while mean and standard deviation were used to describe continuous variables. Multiple linear regression and logistic regression were performed to describe the relationship between psychological resilience and BMI after adjusting for sex, age, marital status, education, smoking status, drinking status, physical exercise frequency, and perceived stress. A significant level of *p* < 0.05 was used to indicate statistical significance in this study.

## 3 RESULTS

### 3.1 Basic characteristics of the study participants

A total of 2445 participants were enrolled, comprising 846 (34.6%) males and 1599 (65.4%) females (Table 1). The average age of the entire sample was 41.09 years (SD = 13.72), while the average BMI was 22.37 kg/m^2^ (SD = 2.96). Notably, the participants exhibited an average resilience score of 3.46 (SD = 0.62), with males demonstrating a mean score of 3.50 (SD = 0.63) and females recording an average score of 3.45 (SD = 0.61).

**TABLE 1:** Basic characteristics of the study participants.

	Men	Women	Total	*p*
	(*N* = 846)	(*N* = 1599)	(*N* = 2445)	
Age				
Mean (SD)	40.96 (13.79)	41.16 (13.68)	41.09 (13.72)	0.773
Median [min, max]	39.00 [17.00, 84.00]	39.00 [17.00, 88.00]	39.00 [17.00, 88.00]	
Age group				
16–35	334 (39.5%)	625 (39.1%)	959 (39.2%)	0.870
36–55	384 (45.4%)	723 (45.2%)	1107 (45.3%)	
≥56	128 (15.1%)	251 (15.7%)	379 (15.5%)	
Marital status				
Separate	4 (0.5%)	2 (0.1%)	6 (0.2%)	<0.001*
Divorce	29 (3.4%)	87 (5.4%)	116 (4.7%)	
Widowed spouse	4 (0.5%)	29 (1.8%)	33 (1.3%)	
Single	227 (26.8%)	350 (21.9%)	577 (23.6%)	
Married	582 (68.8%)	1131 (70.7%)	1713 (70.1%)	
Education				
Middle/high school	216 (25.5%)	377 (23.6%)	593 (24.3%)	0.069
College and undergraduate	539 (63.7%)	1101 (68.9%)	1640 (67.1%)	
Master degree or above	80 (9.5%)	103 (6.4%)	183 (7.5%)	
Primary and below	11 (1.3%)	18 (1.1%)	29 (1.2%)	
Occupation				
establishment	205 (24.2%)	395 (24.7%)	600 (24.5%)	<0.001*
Worker	392 (46.3%)	562 (35.1%)	954 (39.0%)	
Student	38 (4.5%)	53 (3.3%)	91 (3.7%)	
Other	211 (24.9%)	589 (36.8%)	800 (32.7%)	
Annual household income				
0–50,000	118 (13.9%)	188 (11.8%)	306 (12.5%)	0.407
50,000–100,000	212 (25.1%)	391 (24.5%)	603 (24.7%)	
100,000–200,000	218 (25.8%)	428 (26.8%)	646 (26.4%)	
≥200,000	298 (35.2%)	592 (37.0%)	890 (36.4%)	
Smoking status				
Never smoke	503 (59.5%)	1551 (97.0%)	2054 (84.0%)	<0.001*
Smoke every day	162 (19.1%)	12 (0.8%)	174 (7.1%)	
Smoke, but not every day	64 (7.6%)	10 (0.6%)	74 (3.0%)	
Ever smoke	117 (13.8%)	26 (1.6%)	143 (5.8%)	
Alcohol drinking				
Never drink	322 (38.1%)	1222 (76.4%)	1544 (63.1%)	<0.001*
Drink every day	19 (2.2%)	1 (0.1%)	20 (0.8%)	
Drink occasionally	388 (45.9%)	318 (19.9%)	706 (28.9%)	
Ever drink	117 (13.8%)	58 (3.6%)	175 (7.2%)	
Activity				
More than once a month	117 (13.8%)	245 (15.3%)	362 (14.8%)	0.052
Less than once a month	128 (15.1%)	314 (19.6%)	442 (18.1%)	
Once a week	159 (18.8%)	264 (16.5%)	423 (17.3%)	
Twice a week	130 (15.4%)	212 (13.3%)	342 (14.0%)	
3 times a week	95 (11.2%)	170 (10.6%)	265 (10.8%)	
4 times a week	52 (6.1%)	113 (7.1%)	165 (6.7%)	
5 or more times a week	165 (19.5%)	281 (17.6%)	446 (18.2%)	
BMI				
Mean (SD)	23.57 (2.93)	21.74 (2.78)	22.37 (2.96)	<0.001*
Median [min, max]	23.53 [14.61, 33.98]	21.48 [13.89, 33.06]	22.06 [13.89, 33.98]	
Sleep duration				
Mean (SD)	7.07 (1.07)	7.02 (1.11)	7.04 (1.10)	0.513
Median [min, max]	7.00 [2.00, 12.00]	7.00 [1.00, 14.00]	7.00 [1.00, 14.00]	
Psychological resilience score				
Mean (SD)	3.50 (0.63)	3.45 (0.61)	3.46 (0.62)	0.211
Median [Min, max]	3.50 [1.50, 5.00]	3.33 [1.33, 5.00]	3.33 [1.33, 5.00]	
Perceived stress score				
Mean (SD)	37.16 (7.17)	37.35 (7.23)	37.28 (7.21)	0.802
Median [Min, max]	38.00 [14.00, 60.00]	38.00 [16.00, 67.00]	38.00 [14.00, 67.00]	
Obesity category				
Underweight	28 (3.3%)	161 (10.1%)	189 (7.7%)	<0.001*
Normal	441 (52.1%)	1126 (70.4%)	1567 (64.1%)	
Overweight	322 (38.1%)	263 (16.4%)	585 (23.9%)	
Obesity	55 (6.5%)	49 (3.1%)	104 (4.3%)	

There was a statistically significant disparity in BMI categories between men and women (*p* < 0.001), with men presenting a higher BMI of 23.57 kg/m^2^ (SD = 2.93) in contrast to women who exhibited an average BMI of 21.74 kg/m^2^ (SD = 2.78). However, no statistically significant distinctions were observed in terms of age, sleep duration, resilience score, and stress score between the two genders (*p* > 0.05).

### 3.2 Association between resilience score and BMI or BMI category

**FIGURE 1:**
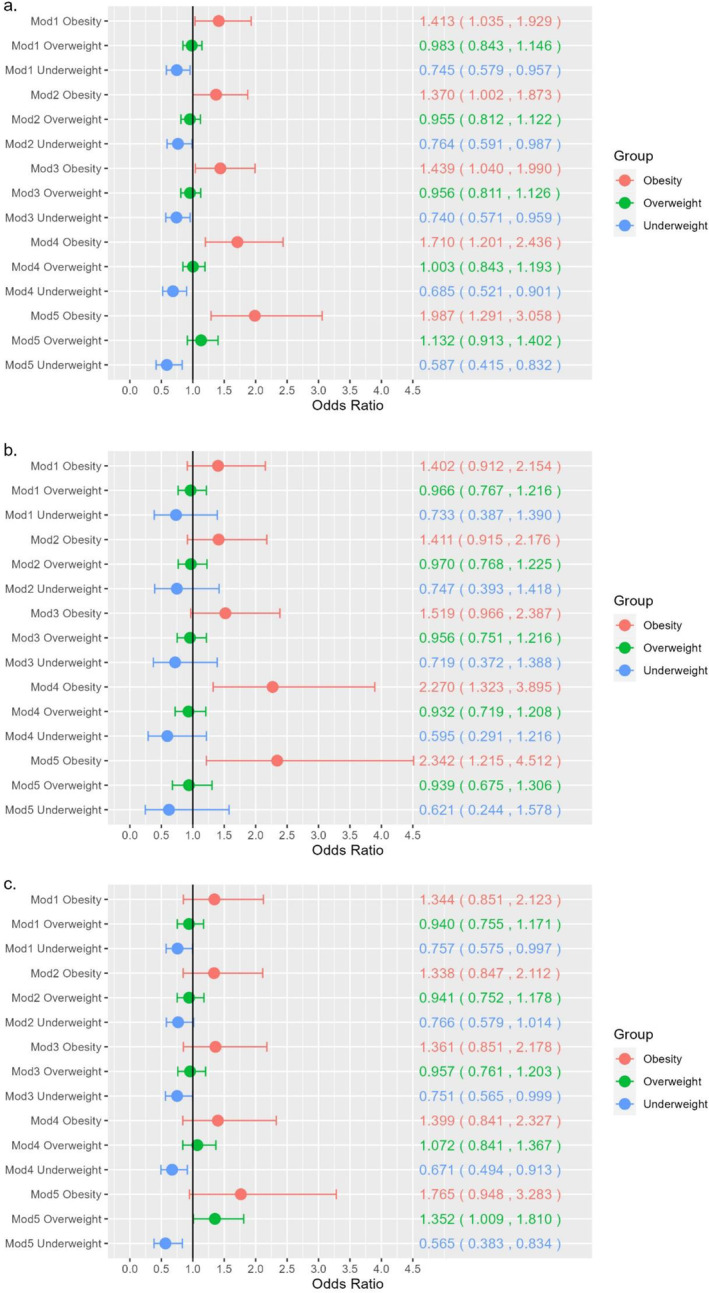
Resilience‐ obesity category forest plot. (A). Resilience‐ obesity category forest plot (total); (B). Resilience‐ obesity category forest plot (men); (C). Resilience‐ obesity category forest plot (women).

In the multiple linear regression model, after adjusting for multiple covariates, the effect of the resilience score on BMI became statistically significant. This significance was observed after adjusting for physical activity, sleep, disease, and self‐evaluated health. The model, which also included stress score and other demographic variables, revealed that higher resilience scores in the population were associated with higher BMI (*β* = 0.502, *p* = 1.21E‐05). This association was found to be significant in women (*β* = 0.571, *p* = 3.632E‐05), but not in men (Table 2). Notably, the interaction term between gender and psychological resilience was not statistically significant (Table S2 and S3). In the fully adjusted logistic regression model, higher resilience scores in the overall sample were found to be significantly associated with obesity (odds ratio [OR] = 1.987, *p* = 0.002). Likewise, lower resilience scores were associated with underweight (OR = 0.587, *p* = 0.003), particularly in women (OR = 0.565, *p* = 0.004, Table 3 and Figure 1A–C).

**TABLE 2:** Multiple linear regression analysis of psychological resilience on body mass index.

Model	Total		Men		Women	
	β(95% CI)	*p*	β(95% CI)	*p*	β(95% CI)	*p*
1	**0.279 (0.182, 0.376)**	**0.004**	0.271 (0.110, 0.430)	0.092	0.198 (0.085, 0.311)	0.081
2	**0.218 (0.129, 0.307)**	**0.015**	0.274 (0.118, 0.430)	0.080	0.186 (0.078, 0.294)	0.086
3	**0.244 (0.154, 0.334)**	**0.007**	0.271 (0.113, 0.429)	0.085	**0.215 (0.106, 0.324)**	**0.049**
4	**0.348 (0.256, 0.440)**	**1.61E‐04**	**0.389 (0.229, 0.549)**	**0.015**	**0.308 (0.195, 0.421)**	**0.007**
5	**0.502 (0.388, 0.616)**	**1.22E‐05**	0.318 (0.113, 0.523)	0.121	**0.571 (0.433, 0.709)**	**3.632E‐05**

*Note*: Model 1: included Psychological Resilience Score; Model 2: included covariate in Model 1, Sex and Age(In men or women: Model 2: Psychological Resilience Score and Age); Model 3: included covariates in Model 2, Marital status, Education, Annual household income and Occupation; Model 4: included covariates in Model 3, Smoking status, Alcohol Drinking, Activity, Sleep Duration, Health self‐evaluation, Hypertension, Heart disease, Cerebrovascular disease, Diabetes, Pulmonary disease, Cancer, Dry eye syndrome, Periodontal disease and Other chronic diseases; Model 5: included covariates in Model 4 and Perceived Stress Score.

Abbreviation: 95% CI, 95% Confidence Interval.

*p value < 0.05 (two‐sided).

**TABLE 3:** Logistic regression analysis of psychological resilience on body mass index category.

		Total		Men		Women	
Model		OR (95% CI)	*p*	OR (95% CI)	*p*	OR (95% CI)	*p*
**1**	Underweight	**0.745 (0.579, 0.957)**	**0.022**	0.733 (0.387, 1.390)	0.341	**0.757 (0.575, 0.997)**	**0.047**
	Overweight	0.983 (0.843, 1.146)	0.824	0.966 (0.767, 1.216)	0.768	0.940 (0.755, 1.171)	0.582
	Obesity	**1.413 (1.035, 1.929)**	**0.030**	1.402 (0.912, 2.154)	0.123	1.344 (0.851, 2.123)	0.204
**2**	Underweight	**0.764 (0.591, 0.987)**	**0.039**	0.747 (0.393, 1.418)	0.372	0.766 (0.579, 1.014)	0.062
	Overweight	0.955 (0.812, 1.122)	0.574	0.970 (0.768, 1.225)	0.797	0.941 (0.752, 1.178)	0.595
	Obesity	**1.370 (1.002, 1.873)**	**0.048**	1.411 (0.915, 2.176)	0.119	1.338 (0.847, 2.112)	0.212
**3**	Underweight	**0.740 (0.571, 0.959)**	**0.023**	0.719 (0.372, 1.388)	0.325	**0.751 (0.565, 0.999)**	**0.049**
	Overweight	0.956 (0.811, 1.126)	0.588	0.956 (0.751, 1.216)	0.713	0.957 (0.761, 1.203)	0.706
	Obesity	**1.439 (1.040, 1.990)**	**0.028**	1.519 (0.966, 2.387)	0.070	1.361 (0.851, 2.178)	0.198
**4**	Underweight	**0.685 (0.521, 0.901)**	**0.007**	0.595 (0.291, 1.216)	0.154	**0.671 (0.494, 0.913)**	**0.011**
	Overweight	1.003 (0.843, 1.193)	0.975	0.932 (0.719, 1.208)	0.595	1.072 (0.841, 1.367)	0.574
	Obesity	**1.710 (1.201, 2.436)**	**0.003**	**2.270 (1.323, 3.895)**	**0.003**	1.399 (0.841, 2.327)	0.196
**5**	Underweight	**0.587 (0.415, 0.832)**	**0.003**	0.621 (0.244, 1.578)	0.317	**0.565 (0.383, 0.834)**	**0.004**
	Overweight	1.132 (0.913, 1.402)	0.258	0.939 (0.675, 1.306)	0.708	**1.352 (1.009, 1.810)**	**0.043**
	Obesity	**1.987 (1.291, 3.058)**	**0.002**	**2.342 (1.215, 4.512)**	**0.011**	1.765 (0.948, 3.283)	0.073

*Note*: Model 1: included Psychological Resilience Score; Model 2: included covariate in Model 1, Sex and Age(In men or women: Model 2: Psychological Resilience Score and Age); Model 3: included covariates in Model 2, Marital status, Education, Annual household income and Occupation; Model 4: included covariates in Model 3, Smoking status, Alcohol Drinking, Activity, Sleep Duration, Health self‐evaluation, Hypertension, Heart disease, Cerebrovascular disease, Diabetes, Pulmonary disease, Cancer, Dry eye syndrome, Periodontal disease and Other chronic diseases; Model 5: included covariates in Model 4 and Perceived Stress Score.

Abbreviation: 95% CI, 95% Confidence Interval.

*p value < 0.05 (two‐sided).

We also examined whether BMI could affect psychological resilience using linear regression models. The results demonstrated that higher levels of BMI were associated with higher levels of psychological resilience (Table S4) and the interaction term between BMI and gender was not statistically significant (Table S5).

## 4 DISCUSSION

The study uncovered a significant positive association between psychological resilience scores and BMI, which persisted even after controlling for various demographic variables and stress levels. Notably, the strength of this association was independent of perceived stress scores. These results indicate that higher levels of psychological resilience were associated with elevated BMI, whereas lower resilience scores were linked to underweight, especially among females. This highlighted the complex interplay between psychological resilience and weight management, emphasizing the necessity for targeted interventions to address the diverse impact of psychological factors on weight outcomes. Recognizing the gender‐specific differences in these associations is essential for developing tailored approaches to address weight‐related issues.

In comparison to a previous study conducted by Barbara Stewart‐Knox et al.,^26^ which focused on individuals aged 43 years and over, findings presented contrasting results. Stewart‐Knox et al. examined the relationship between psychological resilience and BMI in Great Britain and Portugal, two countries with distinct cultural backgrounds. Their study found that among the Portuguese population, lower resilience was associated with higher BMI, which supported findings from another study conducted on US Military Veterans.^19^ In our study conducted on the Chinese population, a different pattern was observed where higher resilience was associated with higher BMI. These divergent associations may be influenced by cultural factors; for example, the Chinese liked to enjoy food culture. In addition, different religious beliefs may also affect mental resilience and the body's physiological response to stress. In order to fully understand these associations, it is necessary to conduct further exploration in culturally diverse populations to further control dietary factors.

There have also been studies that have explored the opposite influence path to ours.^34^ A population‐based study in 2017 and 2019 including first‐grade students in public schools in Tokyo, Japan (*n* = 7328), suggested that maternal pre‐pregnancy obesity was linked to decreased resilience (coefficient: −3.29) in children aged 6–7 years, a negative correlation indicated. Another study of adolescents aged 12–18 years with obesity also provided evidence,^35^ lower school resilience was an independent predictor of having metabolically unhealthy obesity.

Contrary to our initial hypothesis that increased resilience would mitigate the BMI increase caused by stress, a positive association was discovered. However, psychological resilience exhibited a negative correlation with stress, suggesting that the overall effect through the mental resilience‐stress‐BMI pathway should be negative, so the positive association within the psychological resilience‐BMI pathway may be strong.

Individuals with higher levels of psychological resilience were more likely to maintain higher BMI levels. On one hand, this could be attributed to the positive effects of psychological resilience in coping with stress and adversity, enabling individuals to adopt healthier lifestyles and potentially engage more actively in health behaviors such as diet and exercise. Their body composition may also lean toward higher muscle mass rather than lower fat content, which can contribute to an increased BMI. Physical activity not only directly affected BMI but also potentially reduced stress levels and enhanced psychological resilience. Consequently, this may alleviate stress‐related eating behaviors and minimize fat deposition associated with stress responses, thereby promoting BMI normalization and fostering a positive cycle.^21^, ^22^ On the other hand, individuals with high psychological resilience generally enjoyed better living conditions. In the context of Chinese culinary culture, people tended to enjoy food more and worry less, as the Chinese saying goes “a broad heart leads to a plump body,” which may also contribute to higher BMI. However, this is just a preliminary finding, and further research is needed to confirm the exact mechanisms and significance of this association.

Historically, research on obesity and well‐being has primarily focused on investigating negative psychological factors,^26^ while neglecting the exploration of positive traits that could potentially serve as protective factors against obesity. For instance, stress could lead to elevated cortisol levels and glucocorticoids played a role in regulating food intake and energy expenditure.^36^, ^37^ Resilience could serve as a protective factor against binge eating disorder and may have a protective effect against depression or stress.^38^ Furthermore, it may serve as a protective factor for maintaining a healthy BMI.

The association between resilience and obesity may be intricate and might be influenced by various factors, including genetic, environmental, and psychological aspects.^39^, ^40^, ^41^ Individuals with higher levels of resilience may employ healthier coping strategies to mitigate stress, such as adaptive problem‐solving, emotional regulation, or self‐control.^23^, ^42^ These strategies could have a positive impact on their dietary choices, physical activity levels, and overall well‐being. The central nervous system regulates eating behavior, and psychological stress and resilience could affect appetite. However, further research is needed to fully understand the mechanisms behind this relationship.

Increasing income was an important measure to improve psychological resilience among populations. Maintaining a healthy BMI and good sleep habits is very beneficial for individuals' positive mindsets. Good physical health was the cornerstone of individuals' self‐assessment of health, and positive cognitive evaluations also promoted the development of individuals' psychological resilience. In addition, enhancing interpersonal relationship characteristics related to psychological resilience included factors such as hope, self‐efficacy, and coping ability, which were the cornerstone of improving psychological resilience among populations.^43^

The cross‐sectional design of our study limited the establishment of causality, posing a study design limitation. Uncertainty existed in determining the temporal sequence and pathways of influence within the psychological structure under investigation, potentially introducing collider bias. For example, psychological resilience and BMI were treated as confounding factors in our analyses, which may lead to spurious correlations. Our study, however, also stands out for its pioneering exploration of the correlation between psychological resilience and BMI. Despite uncertainties surrounding the interactions of various psychological constructs with other variables, our research has uncovered unique insights within the cultural landscape of China. Future research avenues could delve into gender disparities and the biological mechanisms underpinning positive associations. Furthermore, the inclusion of individuals across diverse age groups in our study population allows for reliable extrapolation of results.

In summary, our study uncovered a substantial relationship between increased psychological resilience and higher BMI, highlighting the complex interconnection between psychological strength and weight regulation. Interventions directed at socioeconomic status, education, lifestyle behaviors, and physical well‐being could offer the potential for strengthening psychological resilience and promoting healthier weight management. Emphasizing self‐efficacy and coping strategies at the individual level could contribute to balanced weight and overall health outcomes, addressing the worldwide issue of obesity. It is essential to address this matter not only at the individual level but also through the enactment of public health policies focused on enhancing psychological resilience.

## CONFLICT OF INTEREST STATEMENT

The authors declare that there are no financial or nonfinancial competing interests. No conflict of interest has been declared by the authors.

## Supporting information

Supporting Information S1

## References

[1] Chinese Working Group on Obesity . Guidelines for the prevention and control of overweight and obesity in Chinese adults (excerpts). Acta Nutr Sin. 2004:1‐4.

[2] De Lorenzo A , Romano L , Di Renzo L , Di Lorenzo N , Cenname G , Gualtieri P . Obesity: a preventable, treatable, but relapsing disease. Nutrition. 2020;71:110615. 10.1016/j.nut.2019.110615 31864969

[3] Yang XD , Jiang S , Wang G , Zhang R , Zhang J , Zhu JS . Link of obesity and gastrointestinal cancer: crossroad of inflammation and oxidative stress. J Biol Regul Homeost Agents. 2015;29(4):755‐760. 26753635

[4] Wang L , Zhou B , Zhao Z , et al. Body‐mass index and obesity in urban and rural China: findings from consecutive nationally representative surveys during 2004‐18. Lancet (London, England). 2021;398(10294):53‐63. 10.1016/s0140-6736(21)00798-4 34217401 PMC7617101

[5] Reynolds K , Gu D , Whelton PK , et al. Prevalence and risk factors of overweight and obesity in China. Obesity. 2007;15(1):10‐18. 10.1038/oby.2007.527 17228026

[6] Kumar R , Rizvi MR , Saraswat S . Obesity and stress: a contingent paralysis. Int J Prev Med. 2022;13:95. 35958362 10.4103/ijpvm.IJPVM_427_20 PMC9362746

[7] Urbanetto JS , Rocha PSD , Dutra RC , Maciel MC , Bandeira AG , Magnago TSBS . Stress and overweight/obesity among nursing students. Rev Lat Am Enfermagem. 2019;27:e3177. 10.1590/1518-8345.2966.3177 31596412 PMC6781428

[8] Farr OM , Li CR , Mantzoros cS . Central nervous system regulation of eating: insights from human brain imaging. Metabolism. 2016;65(5):699‐713. 10.1016/j.metabol.2016.02.002 27085777 PMC4834455

[9] Stults‐Kolehmainen MA , Sinha R . The effects of stress on physical activity and exercise. Sports Med. 2014;44(1):81‐121. 10.1007/s40279-013-0090-5 24030837 PMC3894304

[10] Carnell S , Benson L , Papantoni A , et al. Obesity and acute stress modulate appetite and neural responses in food word reactivity task. PLoS One. 2022;17(9):e0271915. 10.1371/journal.pone.0271915 36170275 PMC9518890

[11] van Strien T . Causes of emotional eating and matched treatment of obesity. Curr Diabetes Rep. 2018;18(6):35. 10.1007/s11892-018-1000-x PMC5918520 29696418

[12] Geliebter A , Aversa A . Emotional eating in overweight, normal weight, and underweight individuals. Eat Behav. 2003;3(4):341‐347. 10.1016/s1471-0153(02)00100-9 15000995

[13] Dy W , Hetherington MM . Emotions and eating. Self‐reported and experimentally induced changes in food intake under stress. Appetite. 2009;52(2):355‐362. 10.1016/j.appet.2008.11.007 19071171

[14] Lemmens SG , Rutters F , Born JM , Westerterp‐Plantenga MS . Stress augments food 'wanting' and energy intake in visceral overweight subjects in the absence of hunger. Physiol Behav. 2011;103(2):157‐163. 10.1016/j.physbeh.2011.01.009 21241726

[15] Kaur Y , de Souza RJ , Gibson WT , Meyre D . A systematic review of genetic syndromes with obesity. Obes Rev: An Official Journal of the International Association for the Study of Obesity. 2017;18(6):603‐634. 10.1111/obr.12531 28346723

[16] Hall JE , do Carmo JM , da Silva AA , Wang Z , Hall ME . Obesity, kidney dysfunction and hypertension: mechanistic links. Nat Rev Nephrol. 2019;15(6):367‐385. 10.1038/s41581-019-0145-4 31015582 PMC7278043

[17] Tomiyama AJ . Stress and obesity. Annu Rev Psychol. 2019;70(1):703‐718. 10.1146/annurev-psych-010418-102936 29927688

[18] He JW , Wang K , Yan H , Feng chao , Guo JL . Research progress on the relationship between overweight and obesity and mental health in military personnel. Occup Health. 2022;38:2147‐2150.

[19] Stefanovics EA , Edwards LM , Pietrzak RH . Personality and body mass index in U.S. Military Veterans: results from the national health and resilience in Veterans study. Psychiatr Q. 2021;92(3):917‐923. 10.1007/s11126-020-09878-4 33389478

[20] Borinsky S , Gaughan JP , Feldman‐Winter L . Perceived overweight/obesity, low resilience, and body size dissatisfaction among adolescents. Obes Res Clin Pract. 2019;13(5):448‐452. 10.1016/j.orcp.2019.08.002 31474380

[21] Nishimi KM , Koenen KC , Coull BA , Kubzansky LD . Association of psychological resilience with healthy lifestyle and body weight in young adulthood. J Adolesc Health: Official Publication of the Society for Adolescent Medicine. 2022;70(2):258‐266. 10.1016/j.jadohealth.2021.08.006 PMC8792157 34521575

[22] Zach S , Fernandez‐Rio J , Zeev A , Ophir M , Eilat‐Adar S . Physical activity, resilience, emotions, moods, and weight control, during the COVID‐19 global crisis. Isr J Health Pol Res. 2021;10(1):52. 10.1186/s13584-021-00473-x PMC8412401 34474685

[23] Van Meter F , Cicchetti D . Resilience. Handb Clin Neurol. 2020;173:67‐73. 10.1016/b978-0-444-64150-2.00008-3 32958194

[24] Liu JJW , Ein N , Gervasio J , Battaion M , Reed M , Vickers K . Comprehensive meta‐analysis of resilience interventions. Clin Psychol Rev. 2020;82:101919. 10.1016/j.cpr.2020.101919 33045528

[25] Walker FR , Pfingst K , Carnevali L , Sgoifo A , Nalivaiko E . In the search for integrative biomarker of resilience to psychological stress. Neurosci Biobehav Rev. 2017;74(Pt B):310‐320. 10.1016/j.neubiorev.2016.05.003 27179452

[26] Stewart‐Knox B , E Duffy M , Bunting B , Parr H , Vas de Almeida MD , Gibney M . Associations between obesity (BMI and waist circumference) and socio‐demographic factors, physical activity, dietary habits, life events, resilience, mood, perceived stress and hopelessness in healthy older Europeans. BMC Publ Health. 2012;12(1):424. 10.1186/1471-2458-12-424 PMC3432605 22686278

[27] Soer R , Six Dijkstra MWMC , Bieleman HJ , et al. Measurement properties and implications of the brief resilience scale in healthy workers. J Occup Health. 2019;61(3):242‐250. 10.1002/1348-9585.12041 30903648 PMC6499349

[28] Kunzler AM , Chmitorz A , Bagusat C , et al. Construct validity and population‐based norms of the German brief resilience scale (BRS). Eur J Health Psychol. 2018;25(3):107‐117. 10.1027/2512-8442/a000016 32671321 PMC7357822

[29] Xue T . Research on Measurement of Simplified Toughness Scale [Master]. Guizhou Normal University; 2021.

[30] Chen W , Liu J , Roger LG . The reliability and validity of the simplified Resilience Scale in Chinese college students. Chin J Clin Psychol. 2020;28(01):24‐28.

[31] Ren YJ , Li YM , Lu QB , et al. Reliability and validity of the simplified resilience scale in Chinese community elderly. Chin J Clin Psychol. 2021;29:721‐724.

[32] Avcp Y , Aamd S , Jkb F , et al. Perceived Stress Scale: confirmatory factor analysis of the PSS14 and PSS10 versions in two samples of pregnant women from the BRISA cohort. Cad Saúde Pública. 2017;33(12). 10.1590/0102-311x00184615 29267695

[33] Mei XX , Zeng YH , Wang XQ , Li SH , Wu JY , Ye ZJ . The influence of stress perception on emotional distress and the chain mediating role of loneliness and sleep quality in nursing graduate students. Occup Health. 2022;38:2110‐2115.

[34] Terada S , Isumi A , Doi S , Fujiwara T . Association of Maternal Pre‐pregnancy Body Mass Index with Resilience and Prosociality of the Offspring Aged 6‐7 Years Old: A Population‐Based Cohort Study in Japan. European Child and Adolescent Psychiatry; 2023. 10.1007/s00787-023-02209-5 37087710

[35] Li MK , Patel BP , Chu L , Strom M , Hamilton JK . Investigating resilience and its association with stress, anthropometrics, and metabolic health in adolescents with obesity: a pilot study. Psychol Health Med. 2023;28(7):1997‐2006. 10.1080/13548506.2022.2059094 35373663

[36] Hewagalamulage SD , Lee TK , Clarke IJ , Henry BA . Stress, cortisol, and obesity: a role for cortisol responsiveness in identifying individuals prone to obesity. Domest Anim Endocrinol. 2016;56(suppl l l):S112‐S120. 10.1016/j.domaniend.2016.03.004 27345309

[37] van der Valk ES , Savas M , van Rossum EFC . Stress and obesity: are there more susceptible individuals? Curr Obes Rep. 2018;7(2):193‐203. 10.1007/s13679-018-0306-y 29663153 PMC5958156

[38] Mathieu J , Brunaud L , Reibel N , et al. Low resilience in severe obesity: marker of adverse childhood experiences and current psychological disorders. Eat Weight Disord: EWD. 2022;27(8):3507‐3519. 10.1007/s40519-022-01488-2 36209466

[39] Faye C , Mcgowan JC , Denny CA , David DJ . Neurobiological mechanisms of stress resilience and implications for the aged population. Curr Neuropharmacol. 2018;16(3):234‐270. 10.2174/1570159x15666170818095105 28820053 PMC5843978

[40] Gloria CT , Steinhardt MA . Relationships among positive emotions, coping, resilience and mental health. Stress Health: Journal of the International Society for the Investigation of Stress. 2016;32(2):145‐156. 10.1002/smi.2589 24962138

[41] Caspi A , Sugden K , Moffitt TE , et al. Influence of life stress on depression: moderation by a polymorphism in the 5‐HTT gene. Science (New York, N.Y.). 2003;301(5631):386‐389. 10.1126/science.1083968 12869766

[42] Mahmoud NN , Rothenberger D . From burnout to well‐being: a focus on resilience. Clin Colon Rectal Surg. 2019;32(6):415‐423. 10.1055/s-0039-1692710 31686993 PMC6824889

[43] Palacio GC , Krikorian A , Gómez‐Romero MJ , Limonero JT . Resilience in caregivers: a systematic review. Am J Hosp Palliat Care. 2020;37(8):648‐658. 10.1177/1049909119893977 31830813

