# Supplementary material for: Association between psychological resilience and body mass index in a community‐based population: A cross‐sectional study
Source: Obes Sci Pract. 2024 May 11;10(3):e761. doi: 10.1002/osp4.761 (PMC11088450; doi:10.1002/osp4.761)
Supplement: Supplementary file 1 — Supporting Information S1 [file OSP4-10-e761-s001.docx]

**Supplementary Materials**

**Table S1. Factor Loadings of the Scale in Exploratory Factor Analysis (EFA)**

|  | Brief Resilience Scale | | Perceived Stress Scale | |
| --- | --- | --- | --- | --- |
|  | PA1 | PA2 | PA1 | PA2 |
| SS loadings | 2.23 | 1.57 | 4.21 | 2.99 |
| Proportion Var | 0.37 | 0.26 | 0.30 | 0.21 |
| Cumulative Var | 0.37 | 0.63 | 0.30 | 0.51 |
| Proportion Explained | 0.59 | 0.41 | 0.58 | 0.42 |
| Cumulative Proportion | 0.59 | 1.00 | 0.58 | 1.00 |

*Note*: In the reliability test of two psychological scales, Cronbach's Alpha coefficients were both above 0.70 (Cronbach's alpha 1 = 0.70; 95% CI: 0.70-0.74; Cronbach's alpha 2 = 0.79; 95% CI: 0.78-0.80).

Furthermore, the sample data were divided into two halves for split-half reliability testing, with exploratory factor analysis (EFA) and confirmatory factor analysis (CFA) conducted separately. The first half of the sample consisted of 1222 participants, and the second half consisted of 1223 participants. The Kaiser-Meyer-Olkin (KMO) values for the Brief Resilience Scale (BRS) in the first half sample were 0.71, and for the Perceived Stress Scale (PSS) were 0.90, indicating suitability for factor analysis. In the second half sample, the KMO values for the Brief Resilience Scale (BRS) and the Perceived Stress Scale were 0.69 and 0.89, respectively, also indicating suitability for factor analysis. Exploratory factor analysis (EFA) suggested that both scales were better fit by a two-factor structure.

**Table S2. Psychological Resilience on Body Mass Index: Multiple Linear Regression Analysis (Gender-Resilience Interaction Analysis)**

| **Variables** | β(95%CI) | P |
| --- | --- | --- |
| Resilience | 0.597 ( 0.431 , 0.763 ) | 3.22E-04 |
| Resilience*Gender | -0.147 ( -0.332 , 0.038 ) | 0.426 |

**Table S3.** **Logistics Regression Analysis of Psychological Resilience on Body Mass Index Category (Gender-Resilience Interaction Analysis)**

| **Variables** |  | **OR(95%CI)** | **P** |
| --- | --- | --- | --- |
| Resilience |  |  |  |
|  | Underweight | 0.587 ( 0.299 , 1.150 ) | 0.120 |
|  | Overweight | 1.151 ( 0.869 , 1.525 ) | 0.327 |
|  | Obesity | 2.203 ( 1.267 , 3.830 ) | 0.005 |
| Resilience*Gender |  |  |  |
|  | Underweight | 1.001 ( 0.500 , 2.005 ) | 0.997 |
|  | Overweight | 0.975 ( 0.697 , 1.363 ) | 0.881 |
|  | Obesity | 0.820 ( 0.414 , 1.625 ) | 0.570 |

**Table** **S4. Multiple Linear Regression Analysis of Body Mass Index on Psychological Resilience**

| **Model** | **Total** | | **Men** | | **Women** | |
| --- | --- | --- | --- | --- | --- | --- |
|  | β(95%CI) | P | β(95%CI) | P | β(95%CI) | P |
| 1 | **0.012 ( 0.008 , 0.016 )** | **0.004** | 0.012 ( 0.005 , 0.019 ) | 0.092 | 0.010 ( 0.004 , 0.015 ) | 0.081 |
| 2 | **0.011 ( 0.007 , 0.016 )** | **0.015** | 0.013 ( 0.005 , 0.021 ) | 0.080 | 0.010 ( 0.004 , 0.016 ) | 0.086 |
| 3 | **0.012 ( 0.008 , 0.017 )** | **0.007** | 0.013 ( 0.005 , 0.021 ) | 0.085 | **0.011 ( 0.006 , 0.017 )** | **0.049** |
| 4 | **0.017 ( 0.012 , 0.021 )** | **1.650E-04** | **0.019 ( 0.011 , 0.027 )** | **0.015** | **0.015 ( 0.009 , 0.021 )** | **0.007** |
| 5 | **0.016 ( 0.012 , 0.019 )** | **1.211E-05** | 0.009 ( 0.003 , 0.015 ) | 0.121 | **0.019 ( 0.014 , 0.024 )** | **3.632E-05** |
| Abbreviation: 95%CI, 95% Confidence Interval. Model 1: included Psychological Resilience Score;  Model 2: included covariate in Model 1, Gender and Age(In men or women: Model 2: Psychological Resilience Score and Age); Model 3: included covariates in Model 2, Marital status , Education , Annual household income and Occupation;  Model 4: included covariates in Model 3, Smoking status ,Alcohol Drinking , Activity , Sleep Duration ,Health self-evaluation, Hypertension, Heart disease, Cerebrovascular disease, Diabetes, Pulmonary disease, Cancer, Dry eye syndrome, Periodontal disease and Other chronic diseases; Model 5: included covariates in Model 4 and Perceived Stress Score. *P value < 0.05 (two-sided) | | | | | | |

**Table S5. Multiple Linear Regression Analysis of Body Mass Index on Psychological Resilience (Gender-BMI Interaction Analysis)**

| **Variables** | β(95%CI) | P |
| --- | --- | --- |
| BMI | 0.011 ( 0.005 , 0.017 ) | 0.056 |
| BMI*Gender | 0.007 ( 0.000 , 0.014 ) | 0.299 |

**Figure S1 a. Scree Plot of the Brief Resilience Scale**


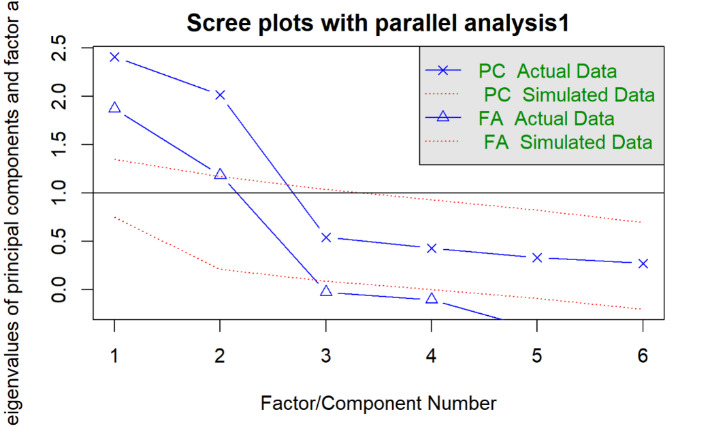


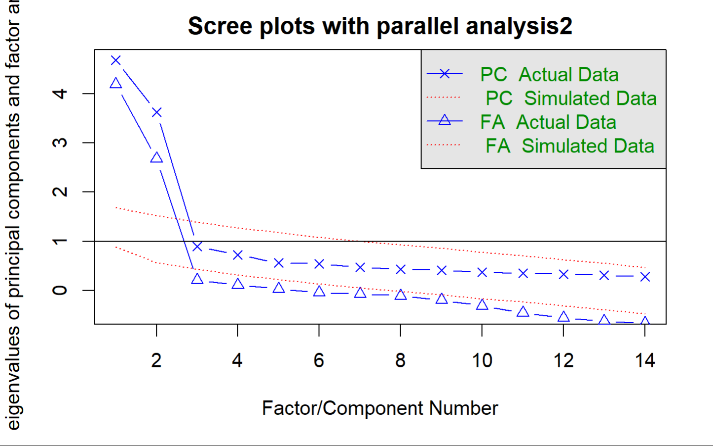
**Figure S1 b. Scree Plot of the Brief Resilience Scale**
